# Effect of Internal and Bulge Loops on the Thermal Stability of Small DNA Duplexes

**DOI:** 10.1021/acs.jpcb.4c03458

**Published:** 2024-10-15

**Authors:** Earle Stellwagen, Paul J. Barnard, Nancy C. Stellwagen

**Affiliations:** † Department of Biochemistry, University of Iowa, Iowa City, Iowa 52242, United States; ‡ Ames High School, Ames, Iowa 50019, United States; § Department of Biochemistry, University of Iowa, Iowa City, Iowa 52242, United States

## Abstract

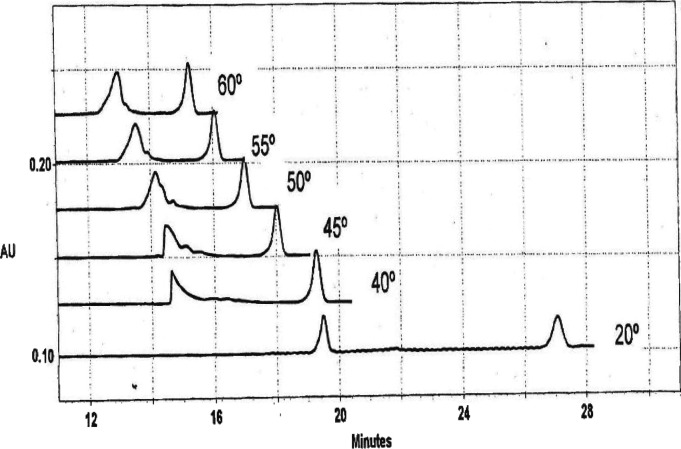

The thermal stabilities of DNA duplexes analogous to the *let-7* microRNA:*lin-41* mRNA complex from *Caenorhabditis elegans* have been measured by free solution capillary electrophoresis. DNA duplexes with the same stems but different types of internal or bulge loops and a control with no loop have also been studied. The melting temperatures of the DNA derivatives increased linearly with the logarithm of the Na^+^ or K^+^ ion concentration in the solution. Peaks in the electropherograms corresponding to duplexes with internal or bulge loops exhibited extensive tailing at high temperatures, suggesting that denaturation occurred by slow exchange between the duplexes and their component single strands. The single strands did not separate completely from the duplexes in aqueous solutions; instead, they appeared as small subpeaks on the tails of the duplex peaks. However, complete separation of the duplexes from their component single strands was observed at 20 °C in solutions containing 300 mM tetrapropylammonium ions. In addition, counterion condensation appears to be significantly reduced in DNA duplexes containing internal or bulge loops.

## Introduction

MicroRNAs (miRNAs) are small, ∼22 nucleotide noncoding RNAs that play an important role in gene regulation by forming complexes with complementary sequences in the 3′-untranslated regions of messenger RNAs (mRNAs).^1−4^ The first two known miRNAs, *lin-41* and *let-7*, were identified in the nematode *Caenorhabditis elegans*, where they were found to regulate the timing of stem-cell division and differentiation. Importantly, the down-regulation of protein-coding genes by miRNA:mRNA complexes has been observed in many plant and animal systems, including humans.^2,4^ Although each miRNA can regulate hundreds of mRNA genes, the mechanism of target selection is not well understood.^5,6^

The physical structures and solution properties of relatively few miRNA:mRNA complexes have been determined to date, although more studies are beginning to appear in the literature. For example, high-resolution NMR studies have been carried out with the *let-7* miRNA:*lin-41* mRNA complexes found in *C. elegans*.^7,8^ One of the constructs characterized in these studies^7^ was a 31-nucleotide duplex with two linear stems bracketing an asymmetric 2 × 3 internal loop that kinked the RNA backbone by ∼20°. One stem of this construct also contained a GU base pair mismatch that is required for biological activity in vivo.^3,9^

Since very little is known about the physical properties of DNA or RNA oligomers containing internal loops, we used free solution capillary electrophoresis (CE) to characterize the thermal stability of the DNA analog of the *let-7* miRNA:*lin-41* mRNA duplex. DNA duplexes containing other types of loops and a control with no loop have also been studied. The DNA analog of the miRNA:mRNA duplex would be expected to have a structure similar to that of its RNA counterpart^10,11^. However, the solution properties of corresponding DNA and RNA oligomers often differ, for reasons that are not clear.^12−14^ The physical properties of DNA oligomers with loops of various types are also of interest because such structures exist during transcription, translation, and protein binding reactions in the cell.^15−17^ In addition, DNA duplexes with internal loops make good intermediates for DNA origami and can be used to design new drugs to treat cancer and other human diseases.^16,18^

CE is a useful technique for studying DNA thermal stability, because DNA hairpins and duplexes have higher charge densities and migrate faster in the electric field than random coils containing the same number of nucleotides.^19−21^ Previous CE studies have shown that the thermal denaturation of DNA hairpins occurs by fast exchange. The electropherograms exhibit a single peak at each temperature, with a mobility that depends on the mole fraction of denatured DNA in the sample at that temperature.^19−22^ By contrast, the results in the present study indicate that the thermal denaturation of DNA duplexes containing internal or bulge loops occurs by slow exchange between the native and denatured conformations. The shapes of the peaks observed during the thermal transitions depend on the type of loop in the duplex, the monovalent cation concentration in the solution and the closeness of the temperature to the melting transition. In addition, counterion condensation appears to be significantly reduced in DNA duplexes containing internal or bulge loops.

## Materials and Methods

### DNA Samples

All DNA oligomers were synthesized by IDT (Integrated DNA Technologies, Coralville, IA), purified by denaturing polyacrylamide gel electrophoresis or HPLC, and analyzed by mass spectrometry. Duplexes were prepared by mixing equimolar quantities of the desired single strands in 10 mM Tris-acetate buffer, pH 8.0, heating at 94 °C for 5 min and cooling slowly to room temperature.^23^ Stock solutions of the duplexes were stored at −20 °C until needed. An oligomer with the sequence ACCTGAT, called ACC7 for brevity, was usually used to monitor the effect of temperature on the viscosity and dielectric constant of water; an oligomer containing 16 thymine residues, called T16, was also used. An aliquot of the desired DNA sample was diluted to 10–50 ng/μL and mixed with the marker immediately before each CE measurement.

The DNA analog of the 31-nucleotide *Let-7* miRNA:*lin-41* mRNA duplex, called mimic for brevity, contained one strand of Let-7 DNA (5′-GGAGGTAGTAGGTCG) and one strand of Lin-41 DNA (5′-CGACCATTGCTGCCTCC). The short names, sequences and types of loops in the various duplexes are given in the first three columns of Table 1. The last three columns summarize the mobility ratios observed in background electrolytes (BGEs) containing 75 mM Na^+^, the melting temperatures (*T*_m_) observed in 75 mM Na^+^; and the number of cations released per phosphate after denaturation (Δn).

**Table 1:** Short Names, Sequences, and Properties of Mimic and Its Derivatives

short name	sequence^a^	loop type	mobility ratio^b^	observed *T*_m_^c^	Δ*n*^d^
mimic	5′-CGACCATTCT**G**CCTCC;	3 × 2	1.30	38	0.14
	3′-GCTGGAT GA**T**GGAGG				
mimic+T	5′-CGACCATTCT**G**CCTCC;	3 × 3	1.29	37	0.14
	3′-GCTGGATTGA**T**GGAGG				
shorty	5′-CGACCCT**G**CCTCC;	0 × 0	1.31	55	0.38
	3′-GCTGGAT**T**GGAGG				
bulge2	5′-CGACCCT**G**CCTCC;	2 × 0	1.31	40	0.14
	3′-GCTGGATGA**T**GGAGG				
bulge3	5′-CGACCATTCT**G**CCTCC;	0 × 3	1.37	37	0.20
	3′-GCTGGGA**T**GGAGG				

a The sequences of the 5'- and 3'- strands of the various derivatives are given, separated by semicolons; the mismatched G and T nucleotides are shown in bold.

b Mobility ratio, duplex/ACC7, measured in 75 mM Na^+^; the variation was ±0.01.

c *T*_m_ measured in 75 mM Na^+^; the reproducibility was ±1 °C.

d Number of cations released per phosphate (see text).

### Buffers

Melting experiments were carried out in background electrolytes (BGEs) containing diethylmalonate as the buffering anion and Na^+^ or K^+^ as the cation. Stock solutions containing 0.5 M diethylmalonic acid [(CH_3_CH_2_)_2_C(COOH)_2,_ Sigma-Aldrich, St. Louis, MO] were titrated to pH 7.3, the p*K*_a_ of the second carboxyl group, with a concentrated solution of NaOH or KOH. Because the second carboxyl group is half ionized at pH 7.3, the cation concentration in each stock solution was 0.75 M; the ionic strength was 1.0 M. To avoid confusion, the buffer concentrations in the following text refer to the cation concentration in the BGE, not the ionic strength of the solution.

### Capillary Electrophoresis

Capillary zone electrophoresis measurements were carried out with a Beckman Coulter (Fullerton, CA) P/ACE System MDQ Capillary Electrophoresis System run in the reverse polarity mode (anode on the detector side) with UV detection at 254 nm, using procedures described previously.^23,24^ The capillaries (Polymicro Technologies, Phoenix, AZ) were 30.9 ± 0.2 cm in length (20.6 ± 0.2 cm to the detector) and 0.75 μm in internal diameter, mounted in a liquid-cooled cassette for good temperature control. The capillaries were internally coated with linear polyacrylamide to minimize the electroosmotic flow (EOF) of the solvent. Previous studies have shown that internal polyacrylamide coatings do not affect the observed mobilities.^25^

The electric field strength ranged from 25 to 175 V/cm, depending on the temperature and the conductivity of the BGE. The current in the capillary was generally less than 60 μA. Under such conditions, Joule heating is negligible^25^ and the mobilities are independent of the applied electric field. The DNA samples were injected hydrodynamically at low pressure (0.5 psi, 0.0035 MPa) for 3 s. The injection volume was ∼22 nL; therefore, the sample plug occupied ∼2.6% of the capillary length. Control experiments showed that the observed mobilities were independent of DNA concentration, the length of the sample plug and the electric field strength.

The free solution mobility of an analyte, μ, is determined^26,27^ by the ratio between its effective charge, *Q*, and its translational frictional coefficient, *f*, as shown in eq 1:
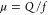
1

For rigid, rod-like molecules such as small DNA oligomers, the translational diffusion constant depends on the axial ratio of the molecule, according to eq 2:
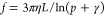
2where *L* is the length of the molecule, η is the viscosity of the solution, *p* is the axial ratio (length/diameter), and γ is a correction for end effects.^28,29^

The observed mobility of an analyte, μ_obs_, is the geometric sum of its intrinsic mobility and the mobility of the solvent due to the electroosmotic flow (EOF). If the EOF mobility is negligible in comparison with the mobility of the analyte, the electrophoretic mobility can be calculated directly from the observed migration times using eq 3:
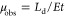
3where μ_obs_ is the observed mobility, *L*_d_ is the distance from the inlet to the detector (in cm), *E* is the electric field strength (in V/cm), and *t* is the time required for the sample to migrate from the inlet to the detector (in seconds).^30^ All mobilities reported here are given in mobility units (m.u.); 1 m.u. = 1 × 10^–4^ cm^2^/(V s); the mobility ratios are dimensionless. The average standard deviation of the mobility ratio measured for a DNA oligomer on any given day was less than ±0.1%; the average day-to-day variation was less than ±0.3%. Such variations are smaller than the sizes of the symbols in the figures below.

### Thermal Melting Studies

Thermal melting studies were carried out by measuring the mobilities of one of the DNA duplexes and ACC7, coinjected into the capillary at the same time in the same solution. The temperature ranged between 15 and 60 °C, the range available on the CE instrument. The capillary was allowed to equilibrate at each temperature for 3 min before a new sample was injected; previous studies had shown that a 3 min wait was sufficient to reach temperature equilibrium.^19−21^ Mobility ratios were calculated at each temperature by dividing the mobility of the DNA duplex by the mobility of the marker, ACC7 or T16. The melting temperatures were independent of which marker was used; however, most studies were carried out with ACC7 because the T16 peak overlapped the duplex peaks at high temperatures.

Plots of the mobility ratios as a function of temperature, called melting curves for brevity, were analyzed by global fits of the melting curves obtained at different cation concentrations to a 4-parameter sigmoid, using the algorithms given in SigmaPlot. The melting curves were assumed to represent two-state conformational transitions that reached a constant mobility ratio at sufficiently high temperatures. The midpoints of the thermal transitions, called the melting temperatures (*T*_m_), were determined from the fits.

The dependence of the melting temperature on the logarithm of the cation concentration, d(*T*_m_)/d(log[cation], can be used to estimate the number of cations released upon denaturation of a DNA duplex.^11,19,31−34^ Since the charge density of a duplex is greater than that of a single-stranded random-coil, some of the cations condensed around a DNA duplex^35^ will be released into the solvent after denaturation. The number of cations released per phosphate, Δn, can be estimated from the dependence of the melting temperature on the logarithm of cation concentration using eq 4:
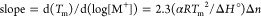
4where [M^+^] is the cation concentration in the solution, *R* is the gas constant, α is a factor that accounts for the dependence of the activity coefficients on concentration, and Δ*H*° is the enthalpy of melting per nucleotide.^11,31,32,34^ Since the factor in parentheses on the right-hand side of eq 4 is not significantly salt- and temperature-dependent,^36^ it can often be set equal to 50,^11,19,31^ giving: Δ*n* ∼ d(*T*_m_)/d(log[M^+^])/115.

## Results and Discussion

### Electrophoretic Mobilities of Mimic and Its Derivatives

**Figure 1:**
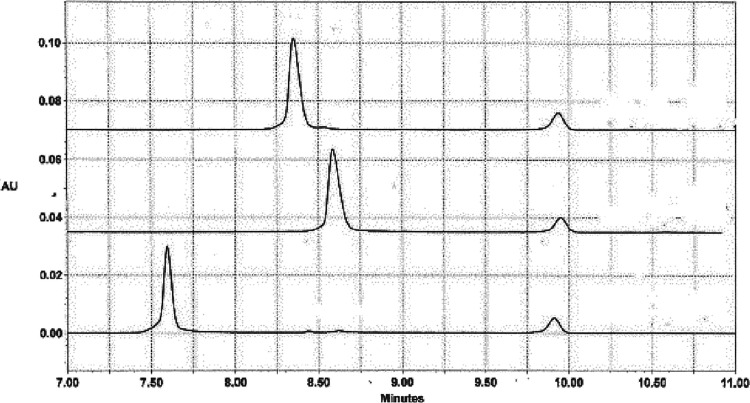
Electropherograms observed for mimic (bottom trace) and its component single strands, Lin-41 (middle trace) and Let-7 (top trace), in 75 mM Na^+^ at 20 °C. The large peaks on the left in each trace correspond to mimic, Lin-41, and Let-7, respectively. The small peaks on the right in each trace correspond to ACC7, added to each solution to monitor changes in the bulk properties of water with temperature. In this and subsequent electropherograms, the absorbance in arbitrary units is plotted as a function of time after injection of the sample into the capillary.

Typical electropherograms observed at 20 °C for mimic and its single stranded components, Let-7 and Lin-41, in 75 mM Na^+^ are illustrated in Figure 1 (bottom to top, respectively). Mimic migrated faster than either Let-7 or Lin-41, as expected since mimic is a duplex and has a higher charge density than its single-stranded components. Interestingly, Let-7, containing 15 nucleotides, migrated faster than Lin-41, with 16 nucleotides, possibly because Let-7 contained 8 guanine residues and one cytosine, while Lin-41 had 2 guanine residues and 8 cytosines. Previous CE studies have shown that small DNA oligomers containing guanine residues migrate faster than oligomers containing the same number of cytosine residues.^37^

The mobility ratios (μ_DNA_/μ_ACC7_) observed for mimic and its derivatives in BGEs containing 75 mM Na^+^ are summarized in column 4 of Table 1. The average mobility ratio observed for mimic, mimic+T, shorty, and bulge2 was 1.31 ± 0.01. The relatively constant mobility ratios observed for these four DNA duplexes suggest that they had similar conformations in solution. By contrast, bulge3 exhibited a mobility ratio of 1.37, suggesting that this oligomer had a different conformation. The bulge3 loop contained three unpaired ATT residues in one strand and no unpaired bases in the other strand. The ATT residues in the bulge3 loop were most likely extruded into the solvent, causing a kink in the oligomer backbone.^38^ Such a conformational change would decrease the axial ratio of the oligomer, decreasing its frictional coefficient and increasing its mobility ratio,^26−29^ as observed. Kinking of the DNA backbone by bulge loops has been observed in a variety of studies.^38,39^ Single molecule FRET experiments have shown that an adenine residue inserted into a DNA duplex causes a bend of ∼32° in the helix backbone.^38,40^ A kink of similar magnitude is predicted for bulge3 by the popular structure-predicting program Mfold.^40,41^

### GT Wobble Base Pair

Mimic and its derivatives contained a mismatched GT base pair in one of its stems, equivalent to the GU mismatch in the RNA sequence^7^ from which mimic was derived. Previous studies of DNA oligomers containing GT mismatches have found that structural perturbations are confined to the mismatch site and the immediately adjacent base pairs.^42^ NMR studies have shown that the electrostatic shielding around mismatched GT base pairs is very similar to that around GC base pairs. The melting temperatures observed for oligomers with corresponding GT and GC base pairs are also very similar.^42^ Hence, the mismatched GT base pair is expected to have relatively little effect on the structure of mimic and its derivatives. However, the rate of base pair opening of mismatched GT base pairs is much faster than observed for GC base pairs.^43^ The increased breathing rate increases solvent exposure, which in turn can decrease the stability of adjacent structural features such as hairpins and internal loops.^44^

### Determination of Thermal Melting Curves

**Figure 2:**
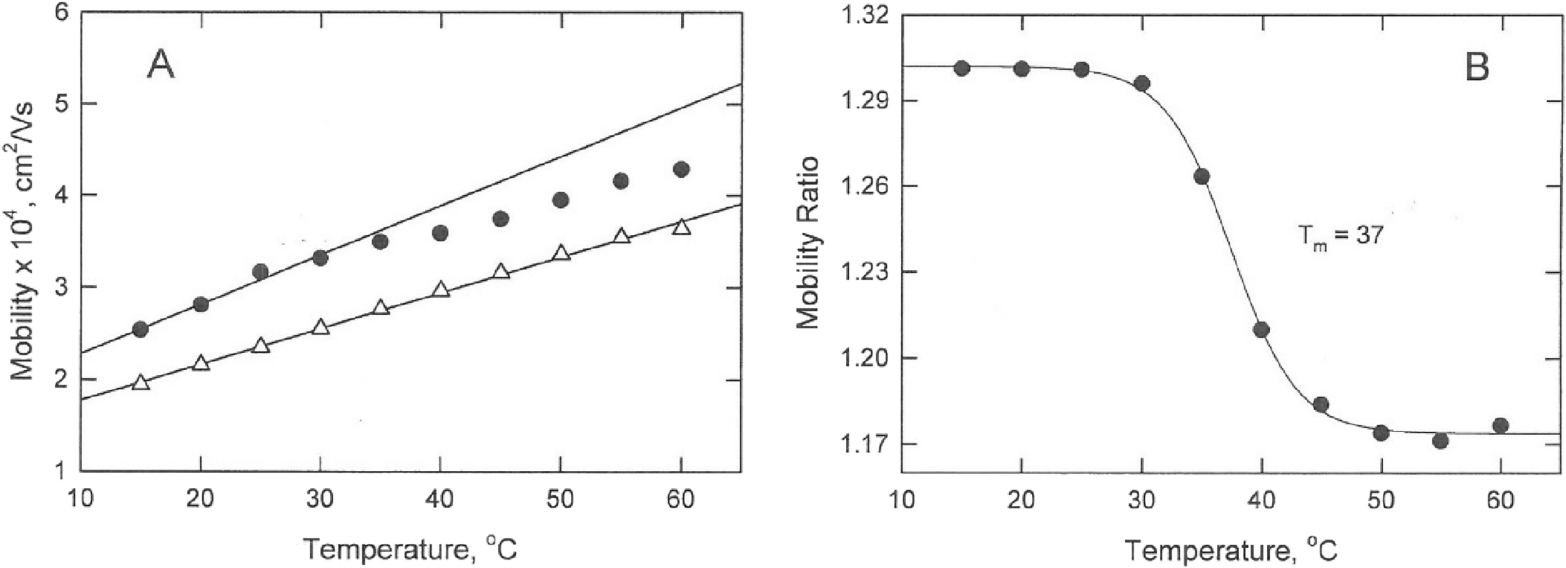
Analysis of DNA thermal transitions observed for mimic in BGEs containing 75 mM Na^+^. (A) Mobilities of mimic (closed circles) and ACC7 (open triangles) plotted as a function of temperature. The solid lines were calculated from eq 5. (B) Mobility ratios, mimic/ACC7, plotted as a function of temperature. The curved line was calculated from the algorithms in SigmaPlot. The melting temperature calculated from the fit was 37 ± 1 °C.

Figure 2A illustrates the effect of temperature on the mobilities observed for mimic and ACC7 in BGEs containing 75 mM Na^+^ ions. The mobility of ACC7 (open triangles) increased linearly with increasing temperature because of the dependence of the viscosity and dielectric constant of water on temperature.^21,22,45^ Since these values are known, the mobility at any temperature, μ_T_, can be predicted from the mobility measured at 20 °C, μ_20_, using eq 5:
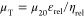
5where ε_rel_ and η_rel_ are the ratios of the dielectric constant and viscosity of water at temperatures *T* and 20 °C. The predicted temperature dependence of the mobility of ACC7 is shown by the lower straight line in Figure 2A. Since the predicted and observed mobilities are equal, ACC7 did not undergo a thermal transition in the investigated temperature range.

Different results were observed for mimic, as also shown in Figure 2A. The mobility of mimic increased linearly with increasing temperature over the range of 15–35 °C, as expected. However, at higher temperatures the observed mobilities became lower than the predicted values, indicating that a thermal transition was taking place. At temperatures of 55 °C and above, the mobility of mimic became parallel to that of ACC7, indicating that the mobilities of both oligomers were now reflecting only the changes in the properties of water with temperature. These temperature-dependent solvent properties can be factored out by dividing the mobility of mimic at each temperature by the mobility of ACC7, measured at the same time in the same solution; the results are shown in Figure 2B. The curved line corresponds to a fit of the data to a four-parameter sigmoid using the algorithms given in SigmaPlot. The midpoint of the thermal transition, called the melting temperature (*T*_m_) for brevity, was 37 ± 1 °C.

### Melting Curves Observed in BGEs with Different [Na^+^]

**Figure 3:**
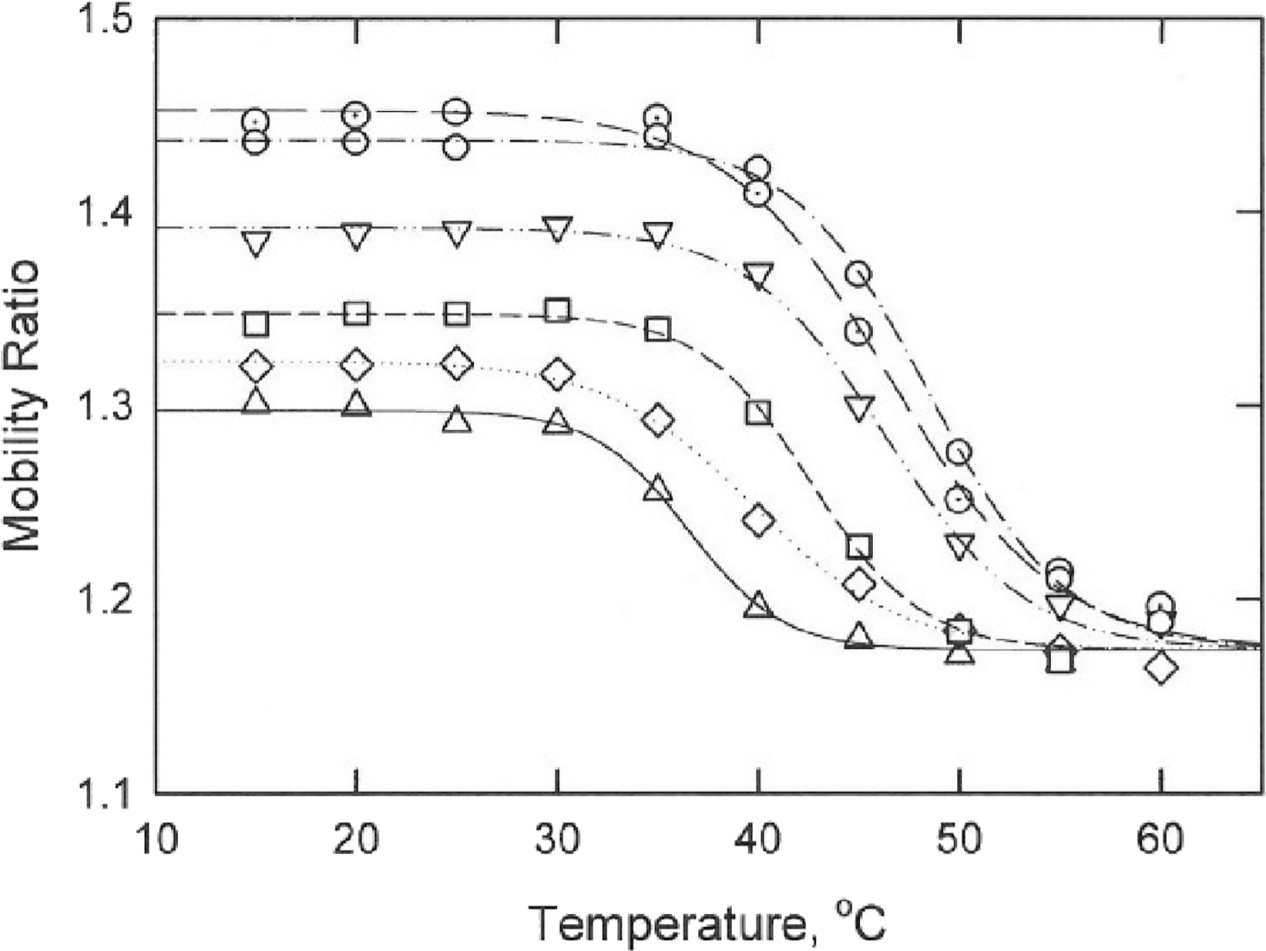
Melting curves observed for mimic+T in solutions with different [Na^+^]. The mobility ratios, μ_mimic+T_/μ_ACC_, are plotted as a function of temperature in BGEs containing 75, 94, 130, 225, 450, and 300 mM Na^+^, reading from bottom to top.

The melting curves observed for mimic+T in BGEs containing 75 to 450 mM Na^+^ ions are illustrated in Figure 3, where the mobility ratios, μ_mimic+T_/μ_ACC7_, are plotted as a function of temperature. The constant mobility ratios observed at temperatures of 25 °C and below indicate that mimic+T was in its native conformation at these temperatures. However, the plateau mobility ratios were not constant but increased with increasing [Na^+^]. Importantly, the EOF mobility has been found to decrease with increasing cation concentration.^46^ Since the observed DNA mobility is the geometric sum of the intrinsic DNA mobility (toward the anode) and the EOF mobility (toward the cathode), the decrease in the EOF mobility with increasing cation concentration would lead to a concomitant increase in the observed DNA mobility. A similar dependence of the plateau mobility on [Na^+^] was observed for all mimic derivatives (not shown).

The mobility ratios observed for mimic+T at various [Na^+^] began to decrease at temperatures above ∼30 °C due to onset of thermal denaturation. The melting curves were relatively sharp, with ∼90% of each transition occurring within ∼15 °C. The mobility ratios appeared to converge to a common value at high temperature, as expected if the random-coil conformations of the denatured components of mimic+T were independent of [Na^+^]. The melting curves were analyzed by a global fit of the data to a four-parameter sigmoid, assuming only that the mobility ratios reached a common value at high temperatures. The curved lines in Figure 3 and the melting temperatures (*T*_m_) given in Table 1 were calculated from the fits. Similar melting curves and identical melting temperatures were obtained if the melting curves were analyzed individually.

### Dependence of the Melting Temperatures on Cation Concentration

**Figure 4:**
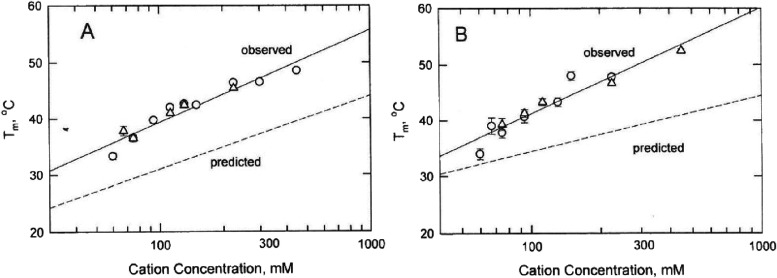
Dependence of the melting temperatures observed for (A) mimic+T and (B) mimic on the logarithm of cation concentration in BGEs containing (open circles) Na^+^ or (open triangles) K^+^ ions. The solid lines were drawn by linear regression; the dashed lines correspond to the melting temperatures predicted for mimic+T and mimic, respectively, by Mfold.^40,41^

As shown in Figure 4A,B, the melting temperatures observed for mimic and mimic+T increased linearly with the logarithm of the cation concentration. Similar results have been observed for other DNA oligomers, using a variety of different experimental methods.^11,21,22,31,32,47^ Since the results observed for mimic and mimic+T were independent of whether the BGE contained Na^+^ or K^+^ ions, the cations interacted in a similar manner with the loops and flanking stems in the DNA duplexes. In addition, the observed melting temperatures were significantly higher than the melting temperatures predicted by the structure-prediction program Mfold,^40,41^ as shown by the dashed lines in Figure 4A,B. The reason for the discrepancy between the observed and predicted melting temperatures is not clear. Presumably, the database from which the Mfold algorithm was derived did not include enough examples of mimic-type sequences to be able to accurately predict the observed melting temperatures. Other important variables that affect the predicted melting temperatures are the types and sizes^21,47,48^ of the cations in the BGE, the ionic strength of the solution,^49^ and the effect of sequence on these parameters.^49,50^

**Figure 5:**
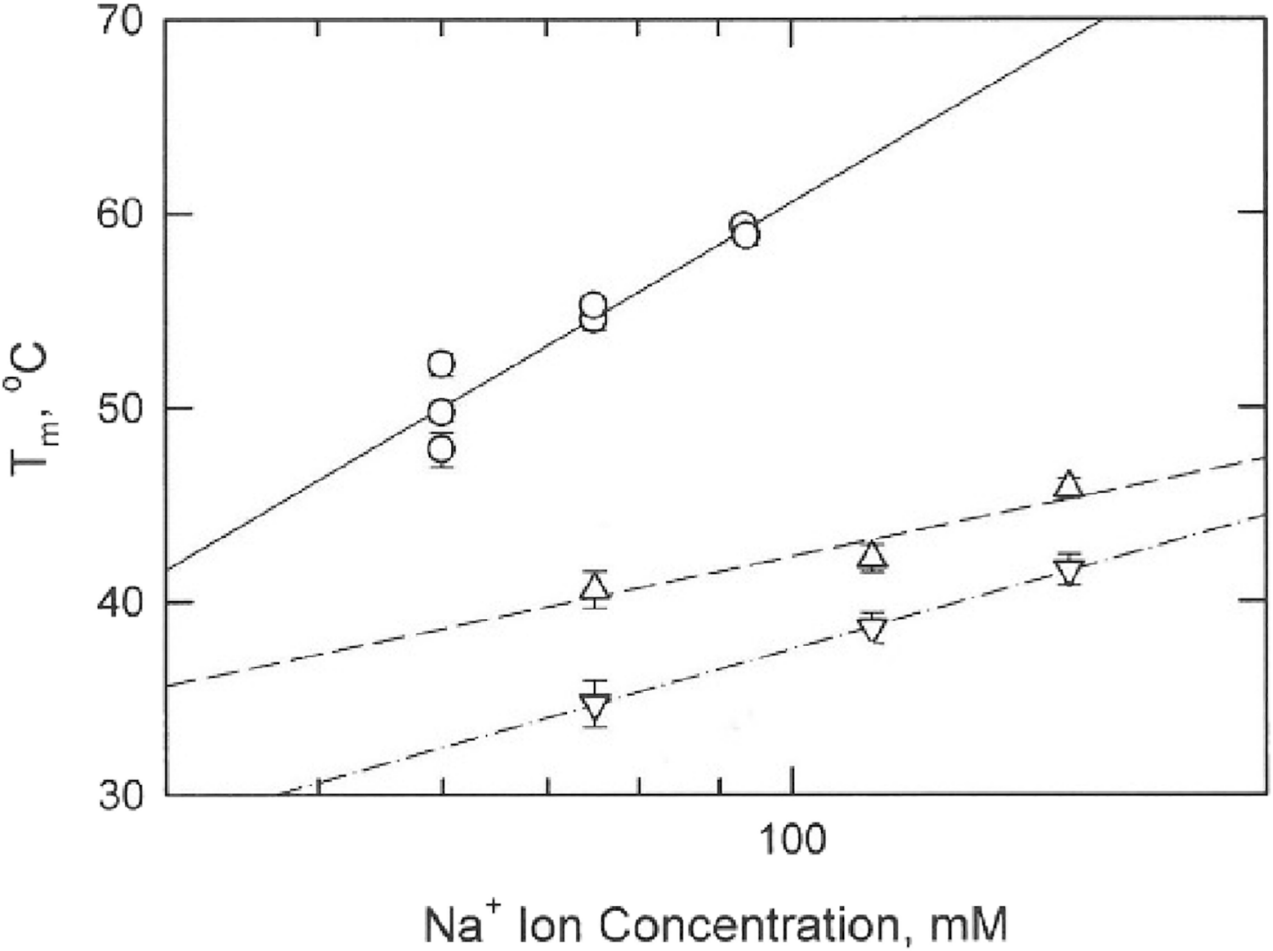
Dependence of the melting temperatures observed for: (open circles) shorty, (open triangles) bulge2, and (open inverted triangles) bulge3 on the logarithm of the Na^+^ ion concentration in the solution.

The melting temperatures observed for shorty, bulge2 and bulge3 also increased linearly with the logarithm of the Na^+^ ion concentration, as shown in Figure 5. The slope of the line observed for shorty, which contained the mimic stems but no bulges, increased more rapidly with the logarithm of [Na^+^] than observed for bulge2 or bulge3.

### Cation Release

The slopes of the lines in Figures 4 and 5, [d(*T*_m_)/d(log[M^+^])], can be used to estimate the number of cations released per phosphate upon denaturation of the parent duplex.^11,31,32^ As shown in the last column of Table 1, mimic, mimic+T and bulge2 released 0.14 Na^+^ ions per phosphate upon denaturation, within the range of values observed for other small DNA duplexes.^11,31−34^ Bulge3 released 0.20 Na^+^ ions per phosphate, suggesting that additional cations were needed to compensate for the high charge density of the phosphate residues near the kink site. Importantly, shorty released 0.38 Na^+^ ions per phosphate upon thermal denaturation, significantly higher than observed for mimic and derivatives containing internal or bulge loops. The combined results suggest that counterion condensation^35^ is reduced significantly if the oligomer contains internal or bulge loops that reduce its effective charge density. Alternatively, or in addition, the discontinuities in base stacking that occur in DNA duplexes containing internal or bulge loops may be relatively easily extended into the flanking stems as the temperature is increased, leading to strand separation at lower temperatures than would be observed for intact DNA duplexes.

### Thermal Denaturation of Mimic and Its Derivatives—Fast Exchange or Slow Exchange?

**Figure 6:**
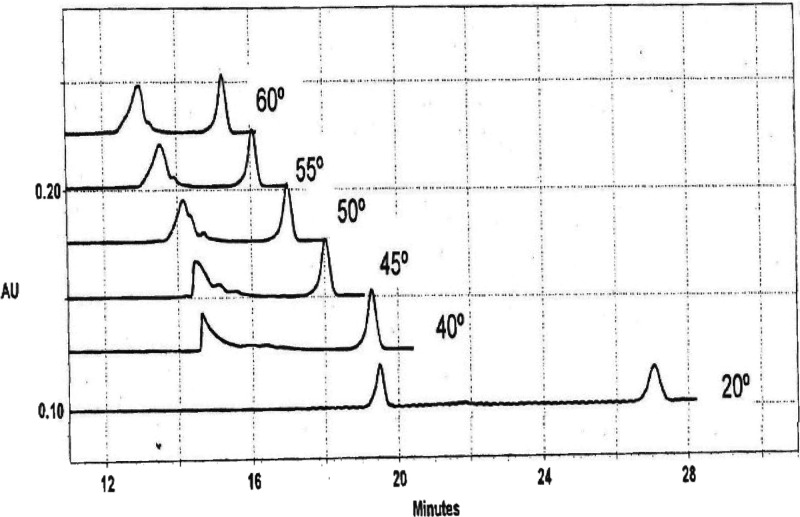
Electropherograms observed for mimic+T at various temperatures in BGEs containing 130 mM Na^+^ ions. The peaks on the left correspond to mimic+T; the peaks on the right correspond to the marker, ACC7.

Because mimic and its single-stranded components exhibit different mobilities in the electric field, it is possible to determine whether thermal denaturation occurs by fast exchange or slow exchange. In fast exchange, DNA hairpins (for example) exhibit a single peak during thermal denaturation, with a mobility that is determined by the mole fraction of denatured DNA present in the sample at that temperature.^19−22^ By contrast, mimic and its derivatives exhibited slow exchange during thermal denaturation, as illustrated in Figure 6 for mimic+T in 130 mM Na^+^. The duplex (left peak in each electropherogram) began to exhibit peak tailing at ∼40 °C, suggesting that some of the components were beginning to separate from the duplex, but the exchange rate was too fast for the single strands to separate completely. By 45 °C, two small subpeaks were seen on the tail of the duplex peak. If the denaturation of mimic+T occurred by an all-or-none mechanism, these subpeaks would correspond to the single-stranded components of mimic+T, Let-7 and Lin-41, which would have migrated more slowly than the duplex in the electric field (see Figure 1). At 50–55 °C, the two small sub- peaks moved closer to the duplex peak and began to coalesce with it. Only a single peak with an irregular shoulder was observed at 60 °C, suggesting that mimic+T and its partially denatured components were approaching the fast exchange regime. The midpoint of the thermal transition, *T*_m_, observed for mimic+T in 130 mM Na^+^ was 43 ± 1 °C, close to the temperature at which the subpeaks began to be observed in the electropherograms.

Alternatively, mimic+T could have melted in a stepwise manner, if the long and short arms surrounding the internal loop melted at different temperatures. In such a case, the faster subpeak (closer to the duplex maximum) would correspond to the long arm (8 bp) and the slower subpeak to the short arm (5 bp), because the amplitudes and mobilities of small DNA oligomers increase with increasing chain length.^25,51^ Sequential melting of the long and short arms would be expected for mimic as well as mimic+T, because both oligomers contained internal loops. However, it is difficult to rationalize the sequential melting of the corresponding arms of bulge2 and bulge3, which did not contain internal loops, and shorty, which was completely base paired.

Importantly, the electropherograms observed during the thermal denaturation of mimic and its derivatives were very similar. All duplex peaks exhibited extensive tailing with increasing temperature, leading to the formation of subpeaks at sufficiently high temperatures. The relative positions and amplitudes of the subpeaks were variable, depending on the type of loop in the sequence, the cation concentration in the BGE, and the closeness of the temperature to the melting temperature. Bulge2 and bulge3 exhibited subpeaks with the highest amplitudes, suggesting that these duplexes were the least stable; the smallest subpeaks were observed for shorty, which was fully base paired. Because of the similarity of the melting curves of mimic and its derivatives, it seems likely that thermal denaturation occurred by an all-or-non process, decreasing the concentrations of the duplexes and increasing the concentrations of their single-stranded components with increasing temperature. However, the complete separation of the duplexes from their component single strands was not observed in BGEs containing Na^+^ or K^+^ ions.

### Characterization of Mimic in Tetraalkylammonium Ion Solutions

**Figure 7:**
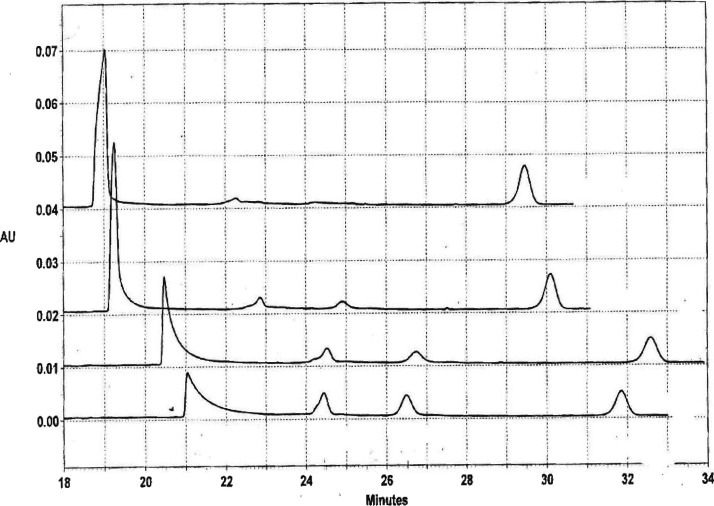
Electropherograms observed for mimic at 20 °C in solutions containing (from bottom to top) 300 mM TPA^+^; 285 mM TPA^+^ plus 15 mM Na^+^; 270 mM TPA^+^ plus 30 mM Na^+^; and 255 mM TPA^+^ plus 45 mM Na^+^. From left to right, the peaks in each electropherogram correspond to mimic, Let-7, Lin-41, and ACC7.

Because the temperature in the capillary could only be increased to 60 °C, it was not possible to characterize the thermal denaturation of mimic and its derivatives at high temperatures and high ionic strengths, where complete strand separation might have been observed. However, high concentrations of tetrapropylammonium (TPA^+^) or tetrabutylammonium (TBA^+^) ions are known to denature DNA at moderate temperatures.^21,22,47,52^Figure 7 illustrates the electropherograms observed for mimic in 300 mM TPA^+^ at 20 °C (lowest trace). Four peaks were observed, reading from left to right: mimic, Let7, Lin-41, and the marker ACC7. Similar results were observed in 300 mM TBA^+^ (not shown). The two small peaks attributed to Let7 and Lin-41 were approximately equal in amplitude, as expected for single-stranded oligomers containing 15 and 16 nucleotides, respectively. If stepwise melting of mimic had occurred, the two small peaks would have differed significantly in amplitude as well as in mobility, since the long and short arms of mimic would have contained 8 and 5 base pairs, respectively. The results therefore confirm that mimic was denatured by an all-or-none process and that baseline separation between the duplex and its component single-strands could be observed at 20 °C in 300 mM TPA^+^. The relative areas of the duplex and monomer peaks in Figure 7 suggest that the mimic duplex was ∼50% denatured in 300 mM TPA^+^ at 20 °C.

Upon adding Na^+^ ions to the BGE (and decreasing the [TPA^+^] to keep the total cation concentration constant), the Let-7 and Lin-41 peaks decreased in amplitude while the duplex peak increased, as shown in the three upper traces in Figure 7. Hence, the concentration of the mimic duplex was preferentially increased by adding Na^+^ ions to the BGE, while the concentrations of the component single strands were decreased. The results indicate that TPA^+^ ions could not dissociate mimic into its component single strands if Na^+^ ions comprised more than ∼20% of the total cation concentration in the solution.

Surprisingly, the spacing between the mimic peak and its component single-strands increased with increasing [Na^+^], indicating that the mobility of the duplex was increasing faster than that of its single-stranded components. This effect appears to have been due, in part, to differences in the shapes of the various peaks: a sharpening of the front edges and a decrease in the asymmetry of the trailing edges. Further studies will be needed to understand these effects.

The complete separation of the mimic duplex from its component single strands in TPA^+^ (and TBA^+^) solutions suggests that the alkylammonium ions had formed electrostatic contact pairs with the phosphate residues in the single-stranded Let-7 and Lin-41 oligomers, preventing them from reassociating to form the duplex again immediately after strand separation. Previous studies have suggested that TPA^+^ ions readily form electrostatic contact pairs with the phosphate residues in single-stranded DNAs, because the flexile backbone of the random coil conformation can easily accommodate the bulky alkylammonium ions.^16,21^ In addition, the TPA^+^ ions may have interacted with mimic and mimic+T by forming electrostatic contact pairs with the phosphate residues surrounding their internal loops. Similar electrostatic contact pairs have been observed for other DNAs.^16,21,38,47^

## Concluding Remarks

The CE studies described above have been used to analyze the thermal stability of the DNA analog of the *let-7* miRNA:*lin-41* mRNA duplex in *C. elegans*, along with DNA derivatives containing other types of internal or bulge loops. Free solution capillary electrophoresis is an ideal technique to use for such studies because DNA duplexes and their component single strands migrate with different mobilities in the electric field and exhibit separate peaks in the electropherograms. In addition, the CE experiments require relatively little purified DNA and can be carried out in aqueous solutions containing biologically relevant cation concentrations.

The melting temperatures (*T*_m_) observed for mimic and its derivatives increased linearly with the logarithm of the monovalent cation concentration in the solution, as observed for other DNA hairpins and duplexes, using a variety of experimental methods.^11,21,22,31,32,48^ The melting temperatures observed for mimic and its derivatives were independent of whether the BGE contained Na^+^ or K^+^ ions, suggesting that neither cation was interacting specifically with the internal or bulge loops in the duplexes.

Although the above results were not unexpected, some of the other results obtained in this study have not been reported previously.

1. The mobilities and mobility ratios observed for the native conformations of mimic and its derivatives increased with the increasing cation concentration in the BGE. This result can be attributed to the fact that the EOF mobility decreases with increasing cation concentration.^46^ Since the EOF and DNA mobilities occur in opposite directions, any decrease in the EOF mobility will necessarily cause a concomitant increase in the observed DNA mobilities.
2. Bulge3, which contains a three-nucleotide ATT bulge, appears to have a kinked conformation in solution. The concomitant decrease in the axial ratio decreased its frictional coefficient,^28,29^ leading to a mobility ratio larger than observed for mimic, mimic+T, shorty, and bulge2. The bend angle observed for bulge3 is likely to have been in the range of ∼20–30°, similar to the bend angles observed for DNA oligomers containing 3-nucleotide adenine bulges,^10,38^ the kink in the backbone of the RNA analog of mimic,^7^ and the bend angle predicted for mimic by the structure-prediction program Mfold.^40,41^
3. Analysis of the number of cations released after denaturation suggests that counterion condensation was significantly greater for shorty, which was completely base- paired, than was observed for mimic and derivatives containing internal or bulge loops. The results suggest that the presence of an internal or bulge loop in a DNA duplex makes the duplex more flexible, so that it behaves electrostatically more like single-stranded DNA.
4. The thermal denaturation of mimic and its derivatives with internal or bulge loops appears to occur by slow exchange. The shapes of the duplex peaks and the amplitudes of the subpeaks observed during the thermal transitions varied with the temperature, the type of loop in the duplex and the monovalent cation concentration in the solution. Complete separation of mimic into its component single strands was not observed in BGEs containing Na^+^ or K^+^ ions.
5. Baseline separation between the mimic duplex and its component single strands was observed in 300 mM TPA^+^ at 20 °C. The duplex peak was reduced in amplitude, while two slower-migrating, baseline-separated peaks with equal amplitudes, corresponding to Let-7 and Lin-41, were observed. The relative areas of the peaks suggested that mimic was ∼50% dissociated into its component single strands in 300 mM TPA^+^ at 20 °C.

Although NMR studies^7^ showed that the miRNA:mRNA complex from which the sequence of mimic was derived has a kink in the backbone at the site of the asymmetric internal loop, we found no evidence for the kinking of mimic in aqueous solutions containing Na^+^ or K^+^ ions. It is theoretically possible that the smaller charge density of mimic, compared with that of a fully base-paired analog, might have decreased the mobility ratio enough to balance the increase in the mobility ratio that would have been caused by the compact shape of a kinked structure.^28,29^ However, the mobility ratios observed for mimic, mimic+T, bulge2 and shorty were equal within experimental error, even though the charge densities were different. Hence, charge density had only a minor effect on the mobility ratios observed for mimic and its derivatives. By contrast, the mobility ratio observed for bulge3, which appears to have a kinked backbone, was significantly larger than observed for mimic and the other derivatives, suggesting that the effect of shape outweighed the effect of charge density in the CE experiments.

The present studies thus provide another example of corresponding RNA and DNA oligomers that have similar structures but exhibit different physical properties in solution. Such differences have often been attributed to the greater intrinsic flexibility of DNA,^12,53^ although the reason is not completely clear.^12,13^ The comparisons are also complicated by the fact that the secondary structures of corresponding RNA and DNA oligomers can be affected by differences in ionic strength.^13^ DNA flexibility can also be enhanced by base pair mismatches,^18^ local melting due to the formation of bubbles,^54^ internal loops located near mismatched base pairs^16,38^ and transient intramolecular complexes formed near stacks of complementary sequences.^54,55^

However, another important factor contributing to the different solution properties observed for corresponding RNA and DNA oligomers is that such comparisons often made using different types of measurements to characterize the nucleic acids. In the present case, the CE measurements were carried out in solutions containing ∼20 μM DNA in buffers containing 75–300 mM Na^+^ ions. The NMR experiments used 2.0 mM RNA in buffers containing ∼30 mM Na^+^ ions.^7^ If mimic and its RNA analog existed in a conformational equilibrium between kinked and unkinked structures, the differences in the RNA and DNA concentrations might have favored the kinked conformation in the NMR experiments and the internal loop in the CE experiments. Further studies would be needed to verify this hypothesis.
